# The rising of old foes: impact of lockdown periods on “non-SARS-CoV-2” viral respiratory and gastrointestinal infections

**DOI:** 10.1007/s15010-022-01756-4

**Published:** 2022-01-25

**Authors:** Nicole Maison, Ana Peck, Sabina Illi, Melanie Meyer-Buehn, Erika von Mutius, Johannes Hübner, Ulrich von Both

**Affiliations:** 1 Department for Asthma and Allergy, Dr Von Hauner Children’s Hospital, Ludwig Maximilians University, Lindwurmstr. 4, 80337 Munich, Germany; 2 Institute for Asthma- and Allergy Prevention (IAP), Helmholtz Center Munich, German Research Center for Environmental Health (GmbH), Munich, Germany; 3 Department of Infectious Diseases, Dr Von Hauner Children’s Hospital, LMU University Hospital, Ludwig Maximilians University, Munich, Germany; 4 German Center for Lung Research (DZL), Munich, Germany; 5 German Center for Infection Research (DZIF), Partner Site Munich, Munich, Germany

**Keywords:** COVID-19, Viral infections, Rhinovirus, Pandemic, Impact, Lockdown

## Abstract

**Background:**

During COVID-19-related public health non-pharmaceutical prevention measures, such as social distancing, lockdown periods and use of face masks, a decrease in viral respiratory and gastroenterological infections was observed worldwide. Following discontinuation of preventative measures, a potential increase of respective infections outside of their usual seasons was a matter of concern.

**Method:**

We aimed to illustrate annual distribution of confirmed viral infections between 2017 and 2021 based on 32,506 clinical samples in a German pediatric tertiary care center and to explore the impact of the COVID-19 pandemic on the epidemiology of these infections in children.

**Results:**

While a decrease in overall viral infections was observed during the first and second lockdown period, an extraordinary increase in the number of viral respiratory infections, predominantly caused by human Rhino-/Enterovirus and respiratory syncytial virus (RSV), was observed after relaxation of preventive measures. Notably, Rhino-/Enterovirus infections increased 4-fold (2020 vs. 2019) and 16-fold (2021 vs. 2019). The occurrence of RSV was observed beginning from June to August 2021 and reached an all-time record with a 25- to 50-fold increase in numbers in September and October 2021 in relation to previous pre-pandemic years (2017–2019). In contrast, for non-respiratory viruses (i.e. Rota-/Norovirus), the effect on respective seasonal patterns was only minimal compared to previous years.

**Conclusion:**

The observed increase in respiratory infections in children is worrying and is already causing hospitals to become overburdened. Enhanced vigilance will be key to face clinical challenges due to these epidemiological changes in viral disease patterns in the months to come.

**Supplementary Information:**

The online version contains supplementary material available at 10.1007/s15010-022-01756-4.

## Background

Following the two lockdown periods (March 2020–May 2020; December 2020–March 2021) in Germany, a joint statement of the German Society for Pediatric Infectious Diseases (DGPI) and the Society for Pediatric Pneumology (GPP) warned of a possible RSV and Influenza wave outside the usual season and suggested appropriate preventive interventions including administration of Palivizumab for children at risk, in Germany [1].

A recent report noted the potential impact of clustered infections from RSV on the health care system and vulnerable patient populations [2]. However, the epidemiological course of RSV and Influenza infections in different regions of the world show fairly different patterns potentially due to differences in infection control measures, climate, and seasonality of infections. In addition, the impact of a potentially increased occurrence of other viral infections such as Rhinovirus infections on children with pre-existing pulmonary conditions is also currently unknown. An increase in viral gastrointestinal infections has barely been reported in the literature until today. In these challenging times, point-of-care diagnostic tests (POCT), such as multiplex-PCR systems, have played an increasingly important role in the diagnosis of viral respiratory infections in acute and emergency care and have generated relevant epidemiological data on these clinical entities [3].

**Fig. 1:**
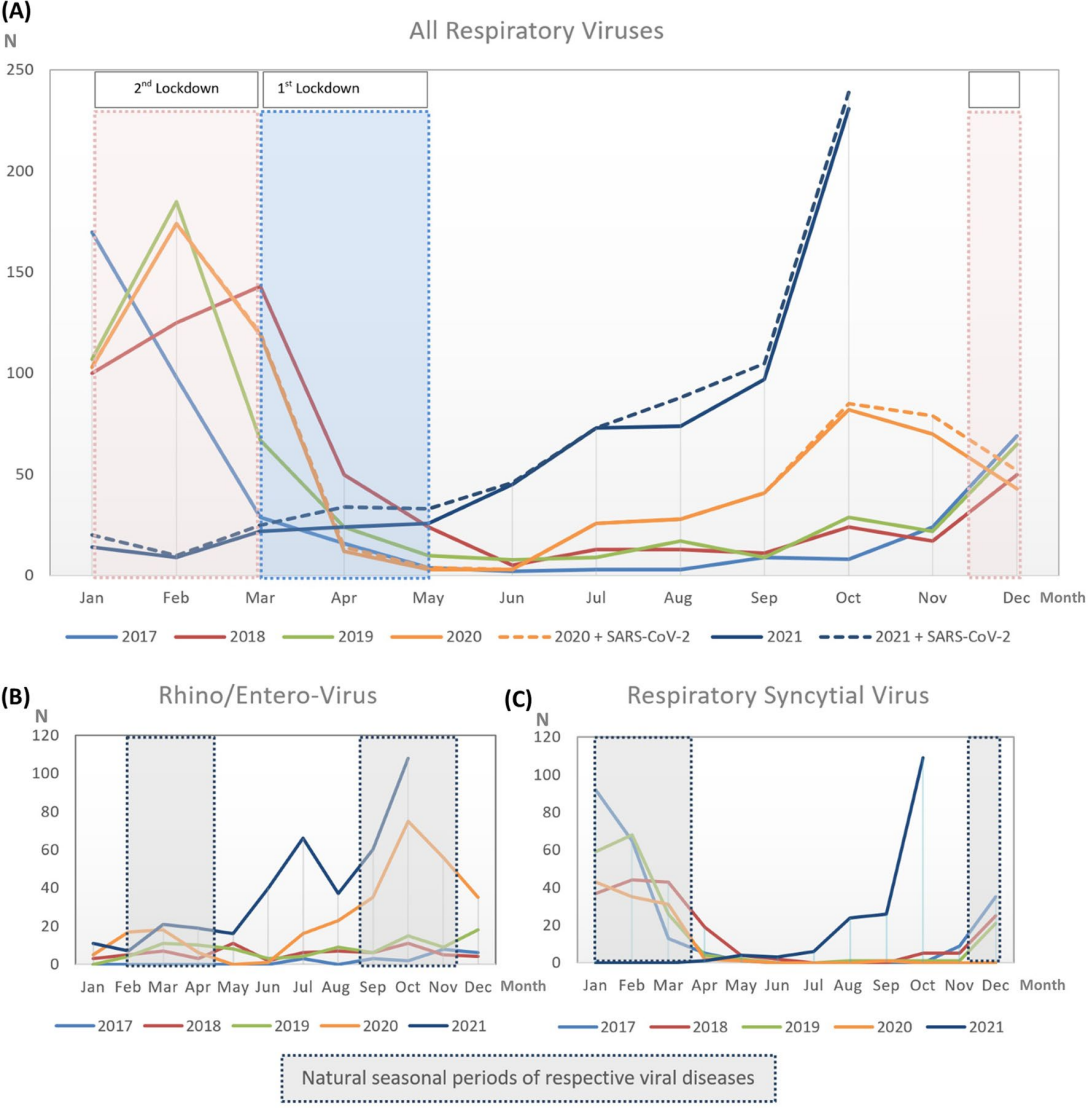
Overview of all respiratory viruses detected between 2017 and 2021. In Germany, the first lockdown was imposed from March 2020 to May 2020, and the second lockdown from December 2020 to March 2021. **A** All respiratory virus detections including influenza A, influenza B, Rhino/Entero-, and respiratory syncytial virus. Adeno-virus from 2017 to 2021. **B** Rhino/Enterovirus detections from 2017 to 2021. **C** Respiratory syncytial virus detection from 2017 to 2021

Besides the current dramatic increase in the number of COVID-19 patients (primarily adult cases) leading to a massive workload for the health system, there is also a concerning shortage of capacities in children’s hospitals. However, in the latter, other viral diseases seem to play more prominent role than SARS-CoV-2 (Fig. 1A).

## Methods

At the Hauner Children's Hospital, a large tertiary care university hospital, the number of accident and emergency (A&E) visits for respiratory or gastrointestinal infections as well as the number of microbiologically detected viral infections in patients between 2019 and August 2021 were investigated. Our analysis is based on results from a total of 32,506 routine clinical specimens of both outpatients and inpatients seen at our hospital between 2017 and 2021. Of these, 3086 (2017 *n* = 476, 2018 *n* = 609, 2019 *n* = 588, 2020 *n* = 756, 2021 *n* = 657) yielded a positive (Table 1) and 29,420 (2017 *n* = 3996, 2018 *n* = 3704, 2019 *n* = 3278, 2020 *n* = 4941, 2021 *n* = 13,501) yielded a negative viral result (Supplementary 1). Until 2020, all tests were carried out in patients with corresponding clinical symptoms following clinical guidelines. In the case of RSV/Rhinovirus, these were presence of an upper and/or lower respiratory-tract infection, age of < 1 year, or presence of a lower respiratory-tract infection with pronounced symptoms in children aged > 1 year. Swabs for Noro-/Rota-virus were taken in case of gastrointestinal infections with severe symptoms that usually required inpatient care. All patients presenting as outpatients or inpatients in the defined study period and receiving a virological test were included in the analysis. The decision as to whether a virological diagnostic test was necessary was made by the attending physician, who followed in-house guidelines. Patients aged 0–18 years were included in the analysis, if they had a positive test result at the in-house microbiology laboratory for at least one of the following viruses: RSV, Rhino-/Entero-, Noro-, Rota-, Adeno-Virus, and Influenza A or B. In cases where two viruses were detected in the same clinical sample, respective viruses were counted individually. With the onset of the COVID-19 pandemic, multiplex-PCR testing (including RSV, Influenza A/B, and SARS-CoV-2) was performed on any patient admitted to our hospital, regardless of clinical symptoms. Therefore, we also calculated the proportion of negative test results on a yearly basis (Supplementary 1). Samples were taken by nasal or pharyngeal swabs (Influenza A/B, RSV, Adeno-virus, and Rhino-/Enterovirus) or via stool sample (Noro/Rota). All samples were tested by immunofluorescence assay (Quidel Sofia 1 or by multiplex-PCR system; Extraction by Nimbus and subsequent analysis by BioRad CFX, Seegene PCR-Kit or BioMerieux Biofire, Askim, SWEDEN, respectively).

**Table 1:** Virus detections between January 2017 and October 2021

	2017	2018	2019	2020	*p* value (2017–2019 vs 2020)	2021	*p* value* (2017–2019 vs 2021)	*p* value# (2020 vs 2021)	Total (*N*/mean)
**Respiratory viruses**									
**Respiratory syncytial virus**									
*N*/%	220/0.46	184/0.30	184/0.31	113/0.15	** < 0.00001**	173/0.26	**0.000895**	** < 0.00001**	874
Age (mean)	1.89	3.03	1.43	1.45	0.05	2.14	0.91	**0.02**	1.99
Sex, *N* (% males)	0.53	0.60	0.61	0.63	0.967752	0.58	0.612598	0.629343	0.58
**Rhino/entero-virus**									
*N*/%	22/0.046	70/0.11	98/0.17	287/0.38	** < 0.00001**	385/0.59	** < 0.00001**	** < 0.00001**	862
Age (mean)	3.53	5.04	4.15	3.88	0.19	4.58	0.69	**0.05**	4.24
Sex, *N* (% males)	0.59	0.51	0.75	0.63	0.833895	0.58	0.583156	0.582855	0.58
**Influenza A**									
*N*/%	145/0.30	179/0.29	175/0.30	136/0.18	** < .000001**	1/0.00	** < 0.00001**	** < 0.00001**	636
Age (mean)	5.54	4.38	4.77	4.47	0.41	9.42	NA	NA	5.72
Sex, *N* (% males)	0.52	0.59	0.51	0.58	0.72668	1.0	NA	NA	0.70
** Influenza B**									
*N*/%	26/0.055	103/0.17	22/0.037	92/0.12	0.06405	1/0.00	** < 0.00001**	** < 0.00001**	244
Age (mean)	4.27	4.97	2.91	5.37	0.16	1.75	NA	NA	3.85
Sex, *N* (% males)	0.30	0.56	0.55	0.67	0.778301	1.0	NA	NA	0.74
** Adeno-virus**									
*N*/%	22/0.046	39/0.064	58/0.10	76/0.10	0.068498	55/0.084	0.436383	0.277027	250
Age (mean)	3.69	3.76	4.8	3.74	0.46	3.58	0.39	0.22	3.91
Sex, *N* (% males)	0.76	0.68	0.60	0.60	0.683359	0.57	0.690973	NA	0.59
**Gastrointestinal viruses**									
**Noro-virus**									
*N*/%	35/0.074	27/0.044	31/0.053	49/0.065	0.487793	36/0.055	0.923463	0.429465	178
Age (mean)	2.96	3.34	3.98	6.74	** < 0.01**	4.68	0.09	0.12	4.34
Sex, *N* (% males)	0.74	0.68	0.57	0.53	0.533599	0.66	0.970145	0.554232	0.59
**Rota-virus**									
*N*/%	6/0.01	7/0.01	20/0.034	3/0.00	**0.006**	6/0.001	0.117633	0.223583	39
Age (mean)	3.18	4.4	2.67	0.22	0.11	3.56	0.74	0.37	2.81
Sex, *N* (% males)	0.50	0.40	0.75	1.0	0.707787	0.56	756,014	NA	0.77
**Total**									
*N*	476	609	588	756	**0.00001**	657	**0.030444**	**0.000196**	3086
Age (mean)	3.58	4.13	3.53	3.70	0.08	4.24	0.46	0.67	3.84
Sex, *N* (% males)	0.56	0.57	0.62	0.66	0.17	0.71	0.6	0.65	0.58

The *p*-values are based on Chi-square test. The bold superscripts indicate for which years numbers of virus detections significantly differ  (*p* < 0.05).

For comparison of proportional incidence of each virus detected 2020 and 2021 in relation to pre-pandemic years (2017–2019).  p* compares 2021 to 2017–2019. *p*# compares 2021–2020. Age comparison was calculated the two-sided *t* test

## Results

During both, the first (March 2020–May 2020) and second lockdown (December 2020–March 2021) in Munich, a decrease in the number of emergency visits and detected viral infections was observed (data not shown). However, after relaxation of NPIs following the first lockdown, numbers of microbiologically confirmed viral infections increased from July 2020 to October 2020 compared to 2017–2019 (Fig. 1A). After the second lockdown in April–May 2021, an unusual surge in the number of patients with respiratory or gastrointestinal symptoms was observed, starting from June to August 2021, with an even more substantial increase in September and October 2021. Analysis of admission diagnoses of respiratory and gastrointestinal infections showed significantly lower numbers during the second lockdown and an increase starting from April 2021 (data not shown).

Comparing the 2020 data with data from previous pre-pandemic years (2017–2019), a significant increase in positive results over all months was observed for RSV, Rhino-/Enterovirus, Noro-virus, and Influenza A, while Influenza B and Rota-virus were not detected more frequently. In 2021 (even when only accounting for the months January–October), the detections of Rhino-/Enterovirus, and Influenza A and B were significantly elevated compared to 2017–2019. Virus detection numbers also varied from 2020 to 2021 showing a significant increase for RSV, Rhino-/Enterovirus, and Influenza A and B. Gender distribution was equal in all viruses between 2017 and 2021. Mean age for RSV and Rhino-/Enterovirus was higher in 2021 compared to 2020, but otherwise stable for other viruses and time points (Table1).

We observed an impressive change in the seasonal pattern of viral infections. The number of all microbiologically confirmed viral infections was significantly lower during the second lockdown from December 2020 until March 2021 but not during the first lockdown. While almost no RSV or Rhino-/Enterovirus infections were detected between July and August in 2019 and 2020, an impressive increase in the number of cases could recently be observed from June to October 2021 (Fig. 1B, C). From April 2021 onwards, the number of any virus detection, including RSV, Rhino-/ Entero- Noro-, Rota-, Adeno-virus, and Influenza A and B, increased disproportionately and reached a peak in October 2021 (2017 *n* = 10, 2018 *n* = 24, 2019 = 33, 2020 *n* = 90, 2021 *n* = 239) (Fig. 1A).

To ensure that the observed notable increase in viral respiratory infections was not due to the increase in testing activity during the COVID-19 pandemic, we carefully analyzed the number of all negative test results stratified by age groups for 2017–2021 (Supplementary 1).

While the epidemiological patterns of respiratory viruses showed impressive changes compared to previous years, number and distribution of gastrointestinal viruses varied only slightly (data not shown). From February to November 2020, hardly any cases of Rota-virus infections were observed at our clinic. However, following the end of the second lockdown, an increase in Rota-virus cases outside the usual season was noted. This was comparable to the number of virus detections in 2019 though.

Noro-virus infections, on the other hand, occurred a bit more frequently in the summer of 2021 compared to previous years.

In addition, and for referencing reasons, we also investigated the number of SARS-CoV-2 infections: 25 children were tested positive in 2020 with a peak from October (*n* = 3), November (*n* = 9), and December (*n* = 9), and 58 children were tested positive until October 2021, with highest numbers in April (*n* = 10) and September (*n* = 14). Thus, SARS-CoV-2 had no significant impact on the observed increase in respiratory-tract infections and played only a minor role in pediatric patients until the end of this analysis (Fig. 1A).

## Discussion

Non-pharmaceutical interventions (NPIs) had an immense impact on the occurrence of viral infections during and after the lockdown periods. While respiratory-tract infections were barely observed during the first and the second lockdown, our data show an overwhelming increase in virus detections particularly for RSV and Rhino-/Enterovirus after relaxation of NPIs. Seasonal patterns of some viruses changed significantly.

Of note, up until August 2021, no off-season peaks of influenza A/B have occurred in our hospital. This might be due to the fact that these infections are vaccine-preventable, so that the impact of SARS-CoV-2 on these viruses may have been limited.

Point-of-care diagnostic tests (POCT), including both antigen-based assays and rapid PCR methods, have been extensively used in pediatric settings [3]. Rapid multiplex-PCR test used for SARS-CoV-2 detection in our setting also included Influenza A/B, RSV, Adeno-virus, and Rhino-/Enterovirus. Although there were significantly more tests performed overall, initially, there was no increase in virus detection. Nevertheless, following reports from Australia, USA, Finland, and France, the predicted increase of RSV infections in the summer was observed here as well, especially in August–October 2021 [4–7]. This is particularly relevant for children younger than 24 months of age, as severe courses of RSV infection are observed at this age. In fact, Amini et al. showed that RSV infections were significantly more severe than Influenza infections in hospitalized children < 3 month [8]. In our tertiary care pediatric center, the rise in viral respiratory infections lead to an increase in hospitalizations due to viral pneumonia with RSV and Rhinovirus being the main triggers [9]. In addition, we observed a higher number of bacterial pneumonia and invasive bacterial infections in our clinical setting (data not shown). This may partially be attributed to reduced natural peer group contacts due to prolonged periods of social distancing measures and closure of institutions of childcare in 2020 and 2021 [10]. Missed opportunities in routine immunizations because of the interruption of standard vaccination schedules may aggravate this development in the months to come. Of note, recent data show that up to 22% of children in Europe have missed a routine immunization visit in 2020 [11].

The increase in microbiologically confirmed RSV and Rhino-/Enterovirus infections since May 2021 was impressive (Fig. 1B). After the first lockdown in 2020, we saw a significant increase in Rhino-/Enterovirus detection in summer and autumn 2020 compared to previous pre-pandemic years, while RSV was barely detected. This might be explained by the natural seasonal period starting earlier for Rhino-/Enterovirus than for RSV: the natural RSV season in Germany was meant to start during the second lockdown. However, both Rhino-/Enterovirus and RSV showed an all-time high in summer of 2021, which might have been caused by an overall reduced immunological competence in the target population due to reduced natural exposure to viruses during the second lockdown period.

Rhinovirus infections in healthy children usually result in only minor respiratory disease. However, in children with recurrent viral induced wheeze and asthma, this virus is the most common trigger for infection-related exacerbations. Recent studies suggest that Rhinovirus strains, type A and C in particular, may have an important impact on the development of asthma [12]. For example, within the multicenter, longitudinal asthma cohort ALLIANCE, it was shown that the decrease in viral infections during the COVID-related lockdown periods had a strong impact on the course of asthma, especially in early childhood, with an improvement of symptoms in up to 30% of children. This was particularly attributed to the decrease in Rhinovirus and RSV infections [13].

So far, little data are available on the duration of immunity to respiratory viruses such as RSV and Rhinovirus. It is assumed that the reduced spread of viruses caused by non-pharmaceutical prevention measures has led to a decrease in herd immunity in the population. According to Sanz-Muñoz et al., this could lead to a shift in seasonal virus infections with significantly higher numbers of cases and longer lasting episodes [14].

In addition to the increase in respiratory infections, we detected slightly more gastrointestinal viruses. In particular for Noro-virus, a trending peak outside the usual season was observed, though to a much lesser degree compared to respiratory infections. Whether this was due to different transmission routes cannot be assessed with certainty and will require further analyses in the future.

A limitation of this study is certainly the monocentric setting and the lack of accurate data on hospitalizations, length of stay, and missing correlation of test results with clinical symptoms. On the other hand, our data comprehensively describe the significant changes in viral infections epidemiology observed in a large tertiary care pediatric center covering a 5-year period (2017–2021) and illustrates the dramatic impact of public health measures on this epidemiology.

## Conclusion

Since the onset of the COVID-19 pandemic and the resulting non-pharmaceutical preventive measures, the epidemiological picture of non-SARS-CoV-2 viral infections has changed fundamentally. The overall rise of old foes—the increase in “non-SARS-CoV-2” viral infections—has a very concerning immediate impact on the care of pediatric patients with a shortage of hospital capacities. More importantly, it may also have long-term consequences on child health in the years to come, since it is still unclear whether the significant increase in Rhino-/Enterovirus infections observed now will lead to an increase in future asthma and wheeze exacerbations. Thus, the observed increase in microbiologically confirmed viral infections following both the first and second lockdown should trigger further investigations and highlights the importance of acquiring natural immunity against common viral infections for children and the collateral effects of the introduction of infection control and social distancing measures.

## Supplementary Information

Below is the link to the electronic supplementary material.

Supplementary file1 (PDF 340 KB)

## References

[1] Huppertz HI, Kopp MV, Hübner J (2021). Prävention von infektionen durch das influenza- und respiratorische synzytialvirus nach aufhebung der lockdown-Maßnahmen. Monatsschr Kinderheilkd.

[2] Burki TK (2021). Circulation of influenza, RSV, and SARS-CoV-2: an uncertain season ahead. Lancet Respir.

[3] Schreiner D, Groendahl B, Puppe W (2019). High antibiotic prescription rates in hospitalized children with human metapneumovirus infection in comparison to RSV infection emphasize the value of point-of-care diagnostics. Infection.

[4] John-Sebastian E, Chisha S, Ruopeng X (2021). Off-season RSV epidemics in Australia after easing of COVID-19 restrictions. medRxiv.

[5] Hodjat P, Christensen PA, Subedi S (2021). The reemergence of seasonal respiratory viruses in Houston, Texas, after relaxing COVID-19 restrictions. Microbiol Spectr..

[6] Haapanen M, Renko M, Artama M (2021). The impact of the lockdown and the re-opening of schools and day cares on the epidemiology of SARS-CoV-2 and other respiratory infections in children—a nationwide register study in Finland. EClinicalMedicine..

[7] Delestrain C, Danis K, Hau I (2021). Impact of COVID-19 social distancing on viral infection in France: a delayed outbreak of RSV. Pediatr Pulmonol.

[8] Amini R (2019). Respiratory syncytial virus contributes to more severe respiratory morbidity than influenza in children < 2 years during seasonal influenza peaks. Infection.

[9] Pagliano P, Sellitto C, Conti V (2021). Characteristics of viral pneumonia in the COVID-19 era: an update. Infection.

[10] Hoch M, Vogel S, Kolberg L (2021). Weekly SARS-CoV-2 sentinel surveillance in primary schools, kindergartens, and nurseries, Germany, June–November 2020. Emerg Infect Dis.

[11] Chiappini E (2021). Impact that the COVID-19 pandemic on routine childhood vaccinations and challenges ahead: a narrative review. Acta Paediatr.

[12] Megremis S (2018). Rhinovirus species-specific antibodies differentially reflect clinical outcomes in health and asthma. Am J Respir Crit Care Med.

[13] Maison N, Herbrüggen H, Schaub B (2021). Impact of imposed social isolation and use of face masks on asthma course and mental health in pediatric and adult patients with recurrent wheeze and asthma. Allergy Asthma Clin Immunol.

[14] Sanz-Muñoz I, Tamames-Gómez S, Castrodeza-Sanz J (2021). Social distancing, lockdown and the wide use of mask; a magic solution or a double-edged sword for respiratory viruses epidemiology?. Vaccines.

