# Supplementary material for: The rising of old foes: impact of lockdown periods on “non-SARS-CoV-2” viral respiratory and gastrointestinal infections
Source: Infection. 2022 Jan 25;50(2):519–24. doi: 10.1007/s15010-022-01756-4 (PMC8787179; doi:10.1007/s15010-022-01756-4)
Supplement: Supplementary file 1 — Supplementary file1 (PDF 340 KB) [file 15010_2022_1756_MOESM1_ESM.pdf]

Supplementary material to:

The rising of old foes – impact of lockdown periods on “non-SARS-CoV-2” viral respiratory and gastrointestinal infections

Age stratified analysis of negative virus test results

The share of positive results of all tests performed was not significantly different in 2017-2020 (11-15% positive results). In 2021, the proportion of positive results was significantly lower at 5%. However, comparing the distribution of negative test results between different age groups, we saw significant differences: while in the age group of 0 - < 3 years the proportion of negative results was stable at 58 - 63% from 2017-2019, it was significantly lower in 2020 with 41% and in 2021 with only 35%, respectively (p-value: <0.0001). In contrast, the proportion of negative results was doubled in the age group from 9 - 12 years (2017-2019: 4-5% versus 2020: 9% and 2021: 10%), and > 12 years (2017-2019: 9-11% versus 2020: 20% and 2021: 24%), as from 2020 onwards, a large number of multiplex PCR tests were performed to rule out SARS-Cov-2 infections rather than because of suspected infection in children older than 6 years. In the age group of 4-6 year-olds, however, no significant difference was observed (Table S1)

Table S1:

Age/ Years	2017	2018	2019	2020	2017-2019 vs 2020	2021	2017-2019 vs 2021	2020 vs 2021	Total
	N/ %	N/%	N/%	N/%	P-Value	N/%	P-Value	P-Value	N/%
0-3 Years	2514/ .63	2157/ .58	1907/ .58	2010/ .41	< 0.00001	4736/ .35	< 0.00001	< 0.00001	13324/ .45
4-6 Years	650/ .16	618/ .17	623/ .19	928/ .19	.062658	2541/ .19	.026692	.951888	5360/ .18
7-9 Years	329/ .08	320/ .09	278/ .08	557/ .11	.000016	1595/ .12	< 0.00001	.31091	3079/ .12
9-12 Years	140/ .04	191/ .05	159/ .05	458/ .09	< 0.00001	1430/ .10	< 0.00001	.008698	2378/ .08
> 12 Years	363/ .09	418/.11	311/ .10	988/ .20	< 0.00001	3199/ .24	< 0.00001	< 0.00001	5279/ .18
Total Negative Results	3996	3704	3278	4941		13501			29420

Table S1: Negative test results from January 2017 – October 2021. For Comparison age related proportion of negative results 2020 and 2021 compared to 2017-2019, p-values were calculated using the Chi square test. (p < .05). P*compares 2021 to 2017-2019, P# compares 2021 to 2020
